# Altered Gut Microbial Diversity and Depletion of SCFA-Producing Taxa Associated with ASD-like Phenotypes in a Prenatal VPA Rat Model

**DOI:** 10.3390/ijms26188931

**Published:** 2025-09-13

**Authors:** Caixia Wu, Xianjie Li, Han Wang, Zhaoming Liu

**Affiliations:** 1 Institute of Biological and Medical Engineering, Guangdong Academy of Sciences, Guangzhou 510316, China; 2 National Engineering Research Center for Healthcare Devices, Institute of Biological and Medical Engineering, Guangdong Academy of Sciences, Guangzhou 510316, China; lixianjiejx@163.com (X.L.); wanghan0603@outlook.com (H.W.); 3 Guangzhou Institutes of Biomedicine and Health, Chinese Academy of Sciences, Guangzhou 510530, China; 4 University of Chinese Academy of Sciences, Beijing 100049, China

**Keywords:** gut microbiota dysbiosis, SCFA-producing taxa, neuroinflammation, prenatal valproic acid exposure, gut–brain axis

## Abstract

Autism spectrum disorder (ASD) involves complex genetic–environmental interactions. Prenatal valproic acid (VPA) exposure, a known environmental risk factor, induces ASD-like phenotypes in rodents, although the mechanisms linking gut microbiota dysbiosis to neurobehavioral deficits remain unclear. Evidence suggests gut–brain axis dysregulation via altered microbial diversity and reduced short-chain fatty acid (SCFA)-producing taxa contributes to ASD pathogenesis. This study investigated whether prenatal VPA exposure drives ASD-like behaviors through gut dysbiosis and SCFA-producer depletion (e.g., Clostridia, Lachnospiraceae), exploring neuroinflammation and oxidative stress as mechanisms. An ASD rat model was established by maternal VPA injection during specific gestational days. Behavioral tests assessed anxiety, sociability, repetitive behaviors, and cognition. Gut microbiota composition (16S rRNA sequencing), cytokine levels (ELISA), oxidative stress markers (biochemical assays), and microglial activation (Iba1 immunofluorescence) were analyzed. VPA-exposed offspring showed ASD-like behaviors accompanied by neurodevelopmental toxicity, manifesting as social deficits, repetitive grooming, and impaired memory. Concurrently, gut analysis revealed reduced alpha diversity and depleted SCFA-producers (e.g., Clostridia, Lachnospiraceae), alongside increased Bacteroidia and Enterobacteriaceae. Neuroinflammation (elevated IL-1β, IL-6, TNF-α, microglial activation) and oxidative stress (reduced GSH, SOD; elevated MDA, NO) were evident. Multivariate analyses linked dysbiosis to behavioral impairments. Prenatal VPA exposure induces gut microbiota dysbiosis, potentially exacerbating neuroinflammation and oxidative stress to drive ASD-like phenotypes. This establishes a mechanistic link between prenatal insults, gut–brain axis disruption, and neurodevelopmental abnormalities, highlighting microbial modulation and SCFA supplementation as potential ASD therapeutics. Furthermore, integrating behavioral, microbial, and molecular analyses advances understanding of gut–brain interactions in ASD and identifies microbiota–metabolite pathways as targets for neurodevelopmental disorders.

## 1. Introduction

Autism spectrum disorder (ASD) is a complex neurodevelopmental disorder characterized by core deficits in social interaction, communication, and repetitive behaviors, with growing evidence implicating bidirectional interactions between the gut microbiota and the central nervous system (gut–brain axis, GBA) in its pathogenesis [1]. Prenatal exposure to valproic acid (VPA), a known teratogen and histone deacetylase inhibitor, has emerged as a validated rodent model to study ASD-like phenotypes, recapitulating behavioral, neurochemical, and immunological abnormalities observed in humans [2]. While previous studies have highlighted gut dysbiosis and altered short-chain fatty acid (SCFA) metabolism in ASD [3], the mechanistic link between microbial diversity loss, SCFA depletion, and neurobehavioral deficits in the VPA model remains incompletely understood.

The gut microbiota plays a pivotal role in neurodevelopment through the production of metabolites such as SCFAs (e.g., acetate, propionate, butyrate), which regulate energy homeostasis, immune function, and blood–brain barrier integrity [4]. In ASD, multiple studies have reported reduced microbial richness and altered SCFA-producing taxa, such as depletion of Clostridia and Lachnospiraceae, alongside enrichment of pro-inflammatory Bacteroidia and Enterobacteriaceae [5]. For instance, in a VPA-induced rat model, reduced alpha diversity (Chao1, Shannon index) and decreased fecal SCFA levels were correlated with increased microglial activation and pro-inflammatory cytokine expression in the prefrontal cortex and hippocampus [6].

Neuroinflammation and oxidative stress are also central to ASD pathogenesis. Prenatal VPA exposure has been shown to induce upregulation of IL-1β, IL-6, and TNF-α, alongside activation of microglia (Iba1+ cells) in limbic brain regions. Concurrently, SCFAs, particularly butyrate, exhibit anti-inflammatory properties and modulate histone deacetylase activity, influencing neuronal plasticity and immune signaling [7]. However, whether SCFA-producing taxa (e.g., Clostridia, Lachnospiraceae) depletion directly drives neuroinflammation or acts as a downstream consequence of microbial dysbiosis remains unclear.

Animal models have further revealed that gut microbiota transplantation from ASD individuals or VPA-exposed rodents can recapitulate ASD-like behaviors, emphasizing the causal role of microbial dysregulation [8]. For example, prebiotic interventions restoring SCFA production have shown promise in ameliorating social deficits in VPA mice, linking microbial metabolism to behavioral outcomes [9]. Despite these advances, the temporal and causal relationships between microbial diversity loss, SCFA depletion, and neurodevelopmental abnormalities in the VPA model require systematic validation.

### 1.1. Existing Studies on the GBA in ASD Suffer from Several Limitations

(1) Causal Mechanism Ambiguity: Most studies establish correlations between gut dysbiosis and ASD symptoms but lack mechanistic clarity. For instance, while reduced SCFA levels are observed in VPA models, whether this is a primary driver of neuroinflammation or a secondary effect of microbial dysbiosis remains unaddressed [10]. (2) Model Translational Limitations: Many rodent studies focus on acute VPA exposure, whereas human ASD risk involves prolonged prenatal exposure. The chronic effects of VPA on microbial-SCFA-neuroinflammation cascades across developmental stages are understudied [11]. (3) Therapeutic Target Gaps: Current interventions targeting the microbiota (e.g., probiotics, prebiotics) lack specificity due to an incomplete understanding of microbial-taxa-SCFA interactions. Identifying key SCFA-producing taxa and their neuroactive metabolites is critical for developing precision therapies [12].

### 1.2. Our Approach to Addressing These Gaps

(1) Mechanistic Dissection: Using 16S rRNA sequencing, we systematically characterize the gut microbiota in VPA-exposed rats, distinguishing causal relationships from correlations. (2) Translational Model Refinement: By employing a clinically relevant VPA dosing regimen (400–450–400 mg/kg on GD11.5–12.5–13.5), we mimic human first-trimester exposure, enhancing model relevance. (3) Multi-Omics Integration: Combining behavioral assays (open field, three-chamber social interaction), neuroinflammation profiling (ELISA, immunofluorescence), and oxidative stress markers, we establish a comprehensive framework linking microbial-metabolite-neuronal pathways.

Prenatal VPA exposure disrupts gut microbial diversity, particularly reducing SCFA-producing taxa, which leads to systemic and central neuroinflammation via impaired histone deacetylase regulation and oxidative stress, ultimately driving ASD-like behavioral phenotypes.

This study, by identifying SCFA-producing taxa as critical mediators, could unlock novel therapeutic strategies, such as targeted microbial supplementation or histone deacetylase modulation, which are more precise than current non-specific interventions. This work not only advances our understanding of ASD etiology but also provides a translational framework for developing microbiota-based therapies.

## 2. Results

### 2.1. Open Field Test Behavioral Analysis

The open field test was employed to evaluate spontaneous exploratory behaviors and anxiety-related responses in experimental animals within a novel environment.

**Figure 1:**
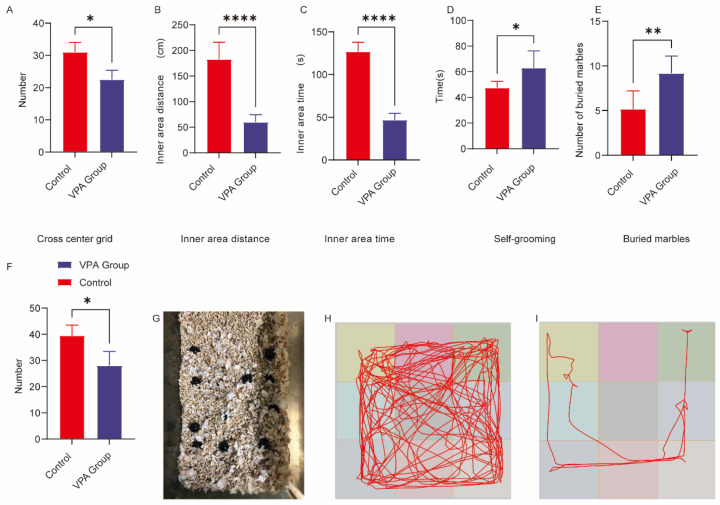
Behavioral analysis of SD rats in the open field and repetitive stereotypic behaviors. (**A**) Cross-center grid; (**B**) inner area distance; (**C**) inner area time; (**D**) self-grooming; (**E**) buried marbles; (**F**) vertical score; (**G**) Representative diagram of buried marbles; (**H**) open field trajectory map of the control group; (**I**) open field trajectory diagram of the ASD model. The data are presented as the mean ± SD ($\;\bar{X}\;$ ± s, *n* = 12). Statistical significance: * *p* < 0.05, ** *p* < 0.01, **** *p* < 0.0001, compared with the control group.

As shown in Figure 1, valproic acid (VPA) exposure significantly suppressed exploratory behaviors while enhancing anxiety-like responses in Sprague–Dawley (SD) rats. The specific findings are detailed below:

#### 2.1.1. Exploratory Behavior Parameters

VPA-exposed SD rats demonstrated significantly reduced exploratory activity compared to controls. This was evidenced by decreased central grid crossings (Figure 1A, *p* < 0.05) and markedly reduced movement distances in the inner zone (Figure 1B, *p* < 0.0001).

#### 2.1.2. Anxiety-Related Behavioral Manifestations

The VPA-exposed group exhibited pronounced anxiety-like behaviors characterized by significantly shorter duration of inner zone activity (Figure 1C, *p* < 0.0001) and reduced vertical exploration scores (Figure 1F, *p* < 0.05). Representative locomotion patterns are illustrated in the trajectory diagrams, with Figure 1H showing control group movement and Figure 1I displaying characteristic ASD model behavior.

### 2.2. Analysis of Repetitive Stereotypic Behaviors

(1) Marble-burying Test

The marble-burying test was employed to evaluate repetitive stereotypic behaviors in rodents. Valproic acid (VPA)-exposed Sprague–Dawley (SD) rats exhibited significantly elevated marble-burying counts compared to controls (Figure 1E, *p* < 0.01). Representative images of marble-burying outcomes are shown in Figure 1G.

(2) Self-grooming Behavior

VPA-exposed rats demonstrated significantly increased self-grooming frequency relative to controls (Figure 1D, *p* < 0.05).

Conclusions:

VPA-exposed SD rats displayed enhanced repetitive stereotypic behaviors, including increased marble-burying and self-grooming activities. These findings suggest neurodevelopmental anomalies associated with basal ganglia-cortical circuit dysfunction.

### 2.3. Three-Chamber Social Behavior Analysis

The three-chamber test evaluated social motivation (0–10 min) and social novelty preference (10–20 min) through timed exploration measurements between unfamiliar conspecifics (Stranger 1/2) and non-social stimuli (empty cages/objects).

- Social Motivation Phase (Sociability, 0–10 min)

**Figure 2:**
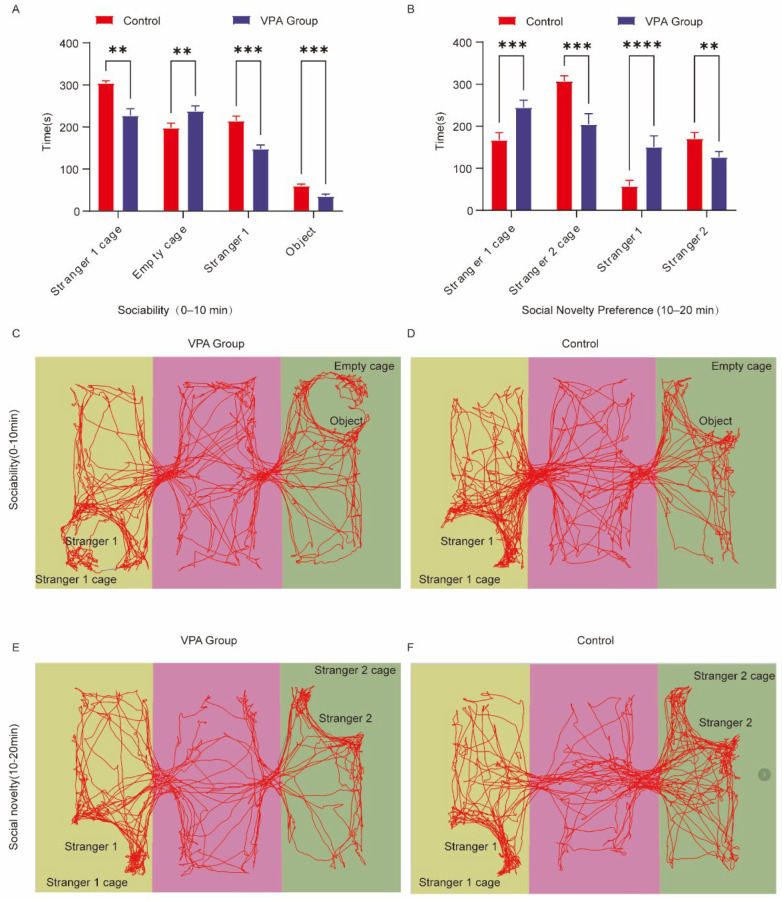
Behavioral analysis of three-chamber social behavior in SD rats. (**A**) Social ability; (**B**) social novelty; (**C**) representative trajectory diagram of social ability (0–10 min) in the ASD model group; (**D**) representative trajectory map of social ability (0–10 min) in the control group; (**E**) representative trajectory diagram of the social novelty preference phase (10–20 min) in the ASD model group; (**F**) representative trajectory diagram of the social novelty preference phase (10–20 min) in the control group. The data are presented as the mean ± SD ($\;\bar{X}\;$ ± s, *n* = 12). Statistical significance: ** *p* < 0.01, *** *p* < 0.001, **** *p* < 0.0001, compared with the control group. Stranger 1 cage: time spent in the Stranger 1 cage; empty cage: time spent in the object cage; stranger 1: interaction time with Stranger 1; object: interaction time with the object; Stranger 2 cage: time spent in the Stranger 2 cage; stranger 2: interaction time with Stranger 2.

VPA-exposed SD rats exhibited impaired social motivation compared to controls (Figure 2A).

(1) Spatial Exploration Preference

The VPA group spent significantly less time exploring the Stranger 1 cage (*p* < 0.01) while displaying increased preference for the empty cage (*p* < 0.01).

(2) Social Interaction Patterns

Reduced sniffing durations were observed in VPA-exposed rats for both Stranger 1 (*p* < 0.001) and objects (*p* < 0.001). Representative movement trajectories are shown in Figure 2C (ASD model) and Figure 2D (control).

Conclusions: VPA-exposed rats demonstrated avoidance of social stimuli (Stranger 1) and preferential exploration of non-social contexts, indicating gestational VPA-induced social competence deficits (*p* < 0.01).

II. Social Novelty Preference Phase (10–20 min, Figure 2B)
(1) Spatial Exploration Dynamics

VPA-exposed rats exhibited prolonged interaction with the familiar Stranger 1 cage (*p* < 0.001) but reduced exploration of the novel Stranger 2 cage (*p* < 0.001).

(2) Social Interaction Bias

Increased sniffing time toward Stranger 1 (*p* < 0.0001) contrasted with diminished investigation of Stranger 2 (*p* < 0.01). Representative trajectories are displayed in Figure 2E (ASD model) and Figure 2F (control).

Conclusions: VPA-exposed SD rats displayed restricted social interest and impaired novelty preference, suggesting compromised cognitive flexibility.

### 2.4. Assessment of Learning and Memory Capacity

The Morris water maze (MWM) task evaluates spatial learning and memory through acquisition trials and probe trials.

#### 2.4.1. Spatial Acquisition Trials

**Figure 3:**
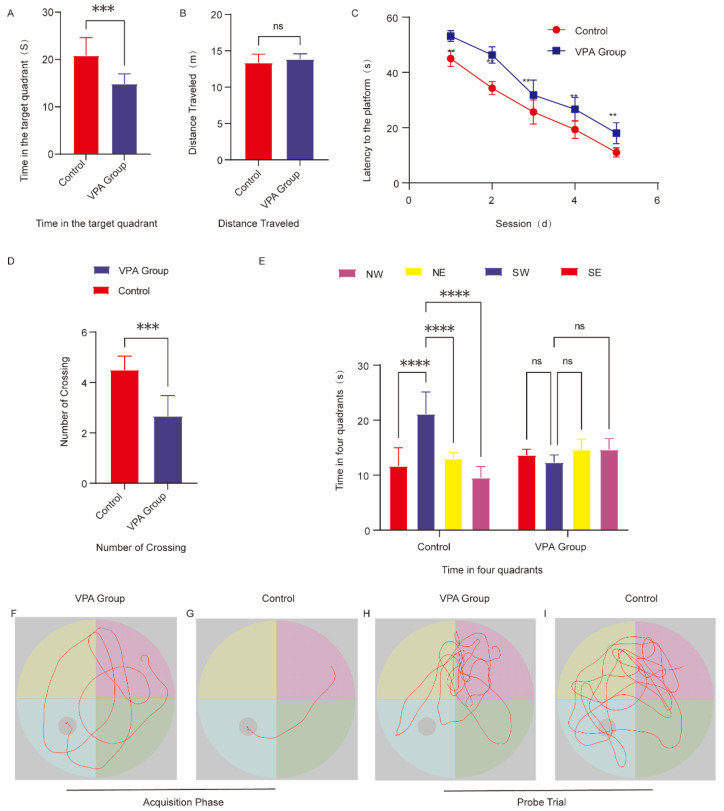
Behavioral analysis of the Morris water maze. (**A**) Time spent in the target quadrant. (**B**) Total swimming distance. (**C**) Escape latency to locate the hidden platform. (**D**) Number of platform crossings. (**E**) Time distribution across the four quadrants during the spatial probe trial. (**F**) Representative swimming paths of the group of SD rats exposed to VPA during spatial acquisition trials. (**G**) Representative Swimming paths of control rats during spatial acquisition trials. (**H**) Representative Exploration trajectories of the group of SD rats exposed to VPA during spatial probe trials. (**I**) Representative Exploration trajectories of control rats during spatial probe trials. The data are presented as the mean ± SD ($\;\bar{X}\;$ ± s, *n* = 12). Statistical significance: ** *p* < 0.01, *** *p* < 0.001, **** *p* < 0.0001, compared with the control group. ns (*p* > 0.05): There is no significant difference.

VPA-exposed SD rats displayed disorganized search patterns during platform localization (Figure 3F), contrasting with the goal-directed navigation observed in controls (Figure 3G). The VPA group demonstrated significantly prolonged escape latencies compared to controls (*p* < 0.01, Figure 3C).

#### 2.4.2. Spatial Probe Trials

Quadrant Preference: VPA-exposed rats showed no target quadrant bias (*p* > 0.05), while controls exhibited significant preference for the target quadrant (*p* < 0.0001, Figure 3E).

Platform Crossings: Reduced platform crossings were observed in the VPA group versus controls (*p* < 0.001, Figure 3D).

Target Quadrant Duration: VPA-exposed rats spent significantly less time in the target quadrant than controls (*p* < 0.001, Figure 3A).

Swimming Distance: No intergroup differences in total swimming distance were detected (*p* > 0.05, Figure 3B), confirming intact motor function.

Representative Trajectories: Control rats focused exploration on the platform quadrant (Figure 3I), whereas VPA-exposed rats exhibited random swimming patterns (Figure 3H).

VPA-exposed SD rats exhibited significant spatial memory deficits characterized by prolonged escape latencies, non-specific search strategies, and impaired target quadrant retention. These cognitive impairments occurred independent of locomotor dysfunction, indicating selective neurocognitive disruption.

### 2.5. Analysis of Neuroinflammatory Markers in the Prefrontal Cortex

**Figure 4:**
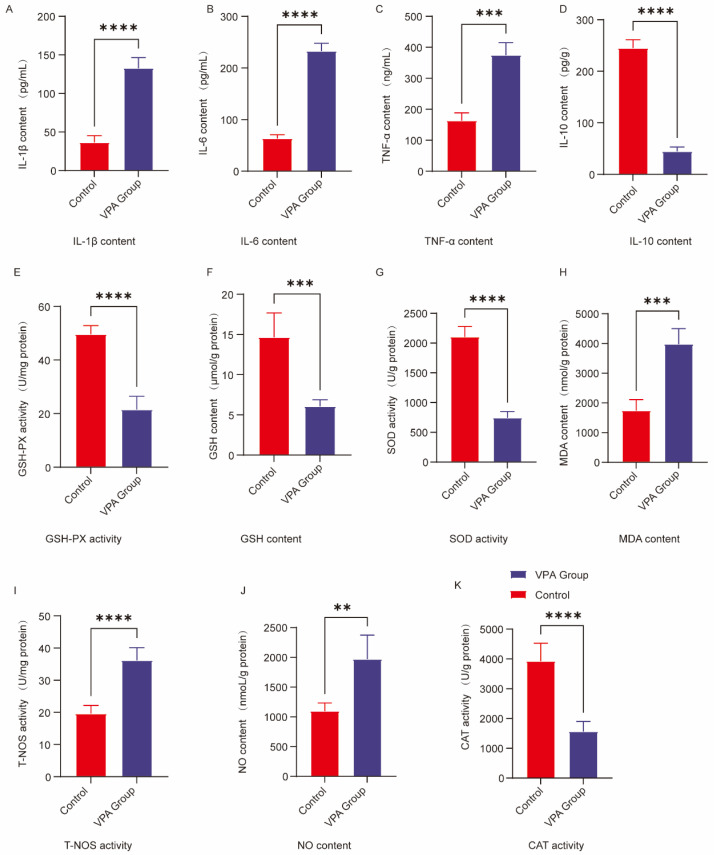
Analysis of neuroinflammatory and oxidative stress levels in the prefrontal cortex. (**A**) IL-1β; (**B**) IL-6; (**C**) TNF-α; (**D**) IL-10; (**E**) GSH-Px activity; (**F**) GSH content; (**G**) SOD activity; (**H**) MDA content; (**I**) T-NOS activity; (**J**) NO content; (**K**) CAT activity. The data are presented as the mean ± SD ($\;\bar{X}\;$ ± s, *n* = 12). Statistical significance: ** *p* < 0.01, *** *p* < 0.001, **** *p* < 0.0001, compared with the control group.

To characterize the neuroinflammatory response in the prefrontal cortex following prenatal VPA exposure, we quantified cytokine profiles using enzyme-linked immunosorbent assays (ELISA). As shown in Figure 4, VPA-exposed Sprague–Dawley rats exhibited a pronounced proinflammatory shift, with significantly elevated levels of interleukin-1β (*p* < 0.0001, Figure 4A), substantially increased concentrations of interleukin-6 (*p* < 0.0001, Figure 4B), and markedly augmented tumor necrosis factor-α expression (*p* < 0.001, Figure 4C) compared to saline-treated controls. Concurrently, we observed significant suppression of the anti-inflammatory cytokine interleukin-10 in VPA-exposed animals (*p* < 0.0001, Figure 4D), indicating a comprehensive disruption of neuroimmune homeostasis. These findings collectively demonstrate that prenatal VPA exposure induces a neuroinflammatory imbalance in the prefrontal cortex through coordinated upregulation of proinflammatory mediators and downregulation of anti-inflammatory signaling pathways.

### 2.6. Analysis of Oxidative Stress Markers in the Prefrontal Cortex

To evaluate oxidative stress responses in the prefrontal cortex following prenatal VPA exposure, we quantified key antioxidant enzymes and oxidative damage markers. As shown in Figure 4, VPA-exposed rats exhibited significant suppression of antioxidant defenses, with markedly diminished glutathione peroxidase activity (*p* < 0.0001, Figure 4E), substantially reduced glutathione concentrations (*p* < 0.001, Figure 4F), significantly lower superoxide dismutase levels (*p* < 0.0001, Figure 4G), and profoundly decreased catalase activity (*p* < 0.0001, Figure 4K). Concurrently, we observed pronounced elevation of oxidative damage indicators, including significantly increased malondialdehyde accumulation (*p* < 0.001, Figure 4H), markedly augmented total nitric oxide synthase activity (*p* < 0.0001, Figure 4I), and substantially elevated nitric oxide concentrations (*p* < 0.01, Figure 4J). These results demonstrate that prenatal VPA exposure induces systemic redox imbalance characterized by compromised antioxidant capacity and exacerbated oxidative damage in the prefrontal cortex.

### 2.7. Microglia Activation and Neuroinflammation in ASD

**Figure 5:**
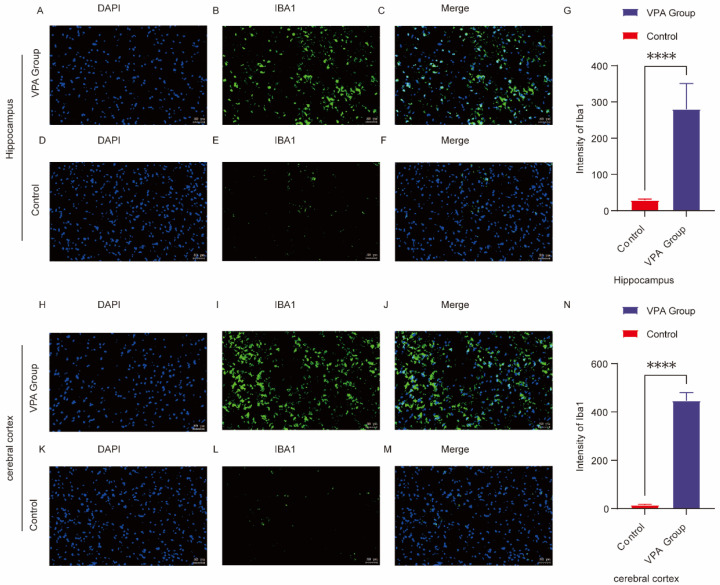
Immunofluorescence of Iba1 in the prefrontal cortex (PFC) and hippocampal CA1 region of control and ASD model rats. (**A**–**F**) Representative immunofluorescence images of Iba1 expression in the hippocampal CA1 region in the control (**D**–**F**) and VPA-exposed (**A**–**C**) groups. (**G**) Mean fluorescence intensity of Iba1, expressed in arbitrary units. (**H**–**M**) Representative Iba1 immunofluorescence in the prefrontal cortex of the control (**K**–**M**) and VPA-exposed (**H**–**J**) groups. (**N**) shows the mean fluorescence intensity of Iba1 in the cerebral cortex, also expressed in arbitrary units. The data are expressed as arbitrary units (AUs) and were analyzed via ImageJ. The values represent the mean ± SD (**** *p* < 0.0001, VPA vs. control; *n* = 6/group). For all the immunofluorescence images, Iba1 is stained green, and DAPI (nuclei) is stained blue. The scale bar in all the images represents 50 µm.

To assess the pathological features of neuroinflammation in the group of SD rats exposed to VPA, the expression level of the microglial marker Iba1 was detected via immunofluorescence staining in this study. As shown in Figure 5A–G, the fluorescence intensity of Iba1 in the hippocampal CA1 region of SD rats was much lower in the control group than in the group of SD rats exposed to VPA (*p* < 0.0001). Similarly, as shown in Figure 5H–N, the fluorescence intensity of Iba1 in the prefrontal cortex of SD rats was much lower in the control group than in the group of SD rats exposed to VPA (*p* < 0.0001). The results revealed that microglial Iba1 expression was significantly elevated in the prefrontal cortex and hippocampal CA1 region of SD rats in the group of SD rats exposed to VPA compared with those in the normal control group.

The above results indicated that the group of SD rats exposed to VPA successfully induced the transformation of microglia from a resting state to a proinflammatory phenotype, suggesting that neuroinflammation may be involved in the pathological process of ASD-related behavioral abnormalities.

### 2.8. Taxonomic and Diversity Shifts in Gut Microbiota of VPA-Induced ASD Rat Models Versus Control Group

**Figure 6:**
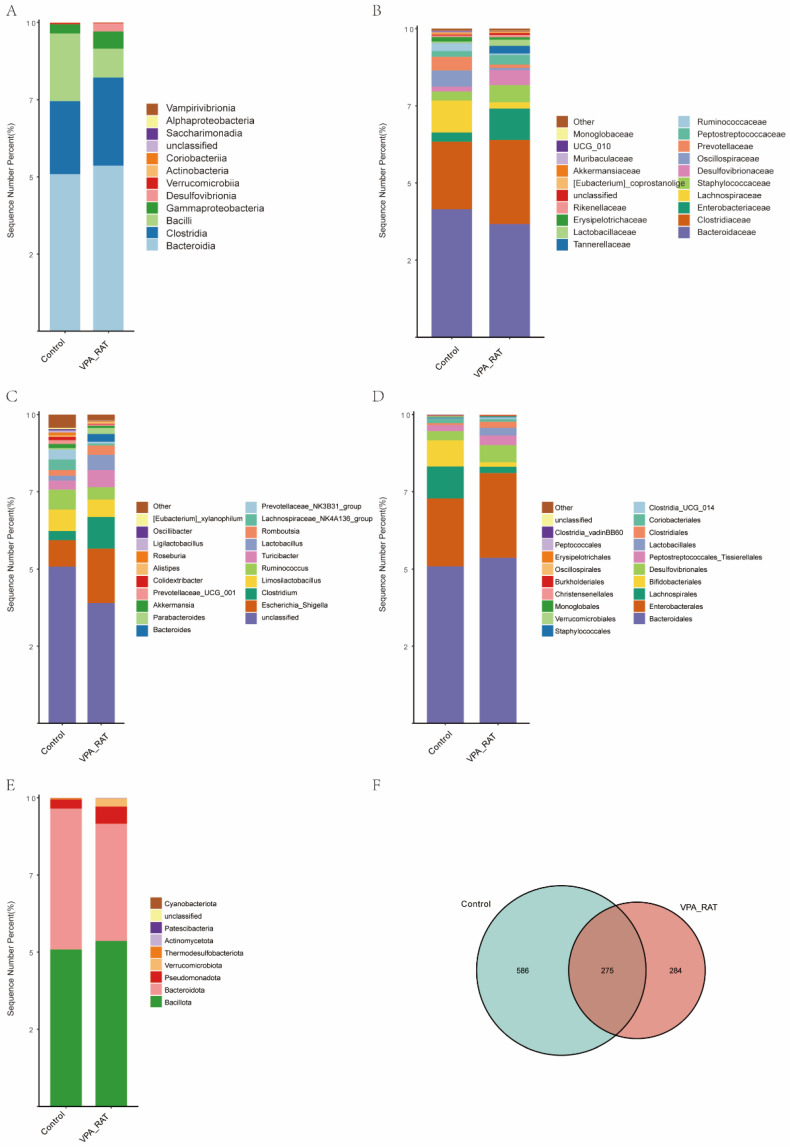
Gut microbiota taxonomic composition and diversity differences between control and VPA-induced ASD rat models. (**A**) Class-level mean abundance barplot. (**B**) Family-level mean abundance barplot. (**C**) Genus-level mean abundance barplot. (**D**) Order-level mean abundance barplot. (**E**) Phylum-level mean abundance barplot. (**F**) Venn plot: Shared and unique OTUs between groups, highlighting reduced microbial overlap in ASD models.

To characterize gut microbiota alterations in the VPA-induced ASD model, we conducted hierarchical taxonomic analysis across multiple levels. As illustrated in Figure 6A, class-level profiling revealed a significant increase in Bacteroidia within the ASD model group compared to controls, suggesting potential dysregulation in carbohydrate metabolism pathways. At the family level (Figure 6B), we observed depletion of SCFA-producing taxa, including Prevotellaceae, Ruminococcaceae, and Lachnospiraceae. The reduction in Prevotellaceae, which is functionally associated with mucin degradation, implies compromised gut mucosal integrity. Furthermore, genus-level composition (Figure 6C) demonstrated diminished abundances of Prevotella and Ruminococcus in ASD models, concomitant with increased Lactobacillus and Escherichia_Shigella. The enrichment of Escherichia_Shigella, a genus linked to pro-inflammatory activity, aligns with the observed inflammatory phenotype. Order-level analysis (Figure 6D) confirmed significant suppression of Bifidobacteriales in ASD animals. At the phylum level (Figure 6E), we documented a marked decrease in Bacteroidota (Bacteroidetes) abundance.

Microbial diversity assessment revealed substantial structural divergence between groups. Venn diagram analysis (Figure 6F) identified 275 shared operational taxonomic units (OTUs), with 586 ASVs unique to controls and 284 unique to ASD models. This reduced taxonomic overlap indicates a distinct microbial community architecture in ASD rats, characterized by depletion of native taxa and emergence of ASD-associated species. Collectively, these hierarchical shifts depict an ASD-associated gut microbiota profile featuring depletion of SCFA-producing taxa (particularly Firmicutes) and reduced microbial diversity, supporting gut dysbiosis as a pathophysiological hallmark that may contribute to the metabolic and immune dysfunction observed in neurodevelopmental disorders.

### 2.9. Gut Microbiota Alterations in VPA-Induced ASD Model Rats

At the family level (Figure S1A): Key taxonomic shifts included elevated Enterobacteriaceae and Erysipelotrichaceae in ASD rats, alongside depletion of Lachnospiraceae and Christensenellaceae.

At the genus level (Figure S1B): Lactobacillus, and Roseburia were reduced in the ASD group.

At the order level (Figure S1C): The ASD group exhibited pronounced taxonomic imbalances. Enrichment of Enterobacterales, Erysipelotrichales, and Staphylococcales contrasted with reduced Lachnospirales, Bifidobacteriales. These opposing trends may reflect disrupted microbial functional networks critical for gut homeostasis.

At the phylum level (Figure S1D): Hierarchical clustering revealed divergent bacterial community structures between the control group and the VPA-induced ASD model rats. Specific bacterial phyla displayed altered relative abundances in the ASD group, as indicated by heatmap color gradients, suggesting a broad reorganization of the gut microbiota. Pseudomonadota and Thermodesulfobacteriota were elevated in the ASD group.

### 2.10. Distinct Gut Microbiota Profiles in VPA-Induced ASD Rat Models Compared to the Control Group

To elucidate taxonomic shifts associated with ASD-like phenotypes, we performed multi-level phylogenetic analyses. The family-level cladogram (Figure S2A) revealed significant enrichment of Peptostreptococcales-Tissierellales (order) and Peptostreptococcaceae (family)—taxa implicated in gut dysbiosis and inflammation—in the VPA-exposed group. Concurrently, Enterobacteriaceae (family) within class Gammaproteobacteria exhibited pronounced elevation, consistent with established patterns of proteobacterial overgrowth in neurodevelopmental disorders. Linear discriminant analysis at the family level (Figure S2B) further identified Gammaproteobacteria (class) and Enterobacterales (order) as dominant discriminators in ASD models, whereas Christensenellaceae and Anaerovoracaceae were enriched in controls. This microbial shift suggests a transition toward pro-inflammatory or opportunistic communities in ASD pathogenesis.

Genus-level characterization (Figure S2C) demonstrated distinct clustering of Enterobacterales and Desulfovibrionales in ASD models, contrasting with Lachnospiraceae predominance in controls. Supporting this, LDA scoring (Figure S2D) confirmed significant enrichment of Escherichia_Shigella and c_Gammaproteobacteria in the ASD group, with the prominence of Escherichia_Shigella aligning mechanistically with dysbiosis-driven inflammatory pathways. At the phylum level, cladogram analysis (Figure S2E) revealed broad structural reorganization marked by significant enrichment of Pseudomonadota—a phylum linked to mucosal colonization and immunomodulation—indicating systemic microbiota restructuring. This finding was corroborated by phylum-level LDA scoring (Figure S2F), where Pseudomonadota exhibited the highest discriminative power between groups, solidifying its role as a biomarker for ASD-associated dysbiosis.

### 2.11. Taxonomic Alterations in Gut Microbiota Composition Between Control and VPA-Induced ASD Model Rats

Comparative analysis of differentially abundant microbial taxa at the family (Figure S3A), genus (Figure S3B), order (Figure S3C), and phylum (Figure S3D) levels.

Family level (Figure S3A): Compared to the control group, VPA-induced ASD model rats exhibited an increased relative abundance of Enterobacteriaceae, while Lachnospiraceae and Ruminococcaceae were significantly reduced. The enrichment of Enterobacteriaceae suggests a potential association with intestinal barrier dysfunction and inflammatory responses. Conversely, the depletion of Lachnospiraceae and Ruminococcaceae—taxa linked to short-chain fatty acid (SCFA) production—may reflect impaired microbial diversity and compromised metabolic homeostasis.

Genus level (Figure S3B): The VPA group showed elevated levels of Escherichia_Shigella, accompanied by reduced abundances of Bifidobacterium. The rise in Escherichia_Shigella implies dysbiosis-associated pathogenicity. The decline in Bifidobacterium aligns with diminished microbial functionality in maintaining gut homeostasis.

Order level (Figure S3C): A marked increase in Enterobacterales and a reduction in Bifidobacteriales were observed in the VPA group. The dominance of Enterobacterales reinforces the trend of pro-inflammatory microbial shifts, while the depletion of Bifidobacteriales highlights a potential loss of beneficial taxa critical for gut immune regulation.

Phylum level (Figure S3D): VPA-treated rats displayed an elevated abundance of Pseudomonadota. The expansion of Pseudomonadota, a phylum containing facultative anaerobes, may indicate environmental stress adaptation in the gut ecosystem.

### 2.12. Gut Microbiota Alpha Diversity Analysis in VPA-Induced ASD Model

Shannon Rarefaction Curve (Figure S4A): The rarefaction curve for the control group (blue) plateaued at higher sequencing depths, indicating robust species richness and evenness. In contrast, the VPA-induced ASD model group (red) reached saturation earlier at lower sequencing depths, with an overall shallower curve, suggesting diminished microbial diversity. This trend implies a less complex and more homogenized gut microbiota structure in the ASD model.

Alpha Diversity Metrics (Figure S4B–F): Consistently, the VPA-induced ASD model group exhibited lower median values across all alpha diversity indices compared to controls (Figures S4B–F). Specifically, the Chao1 index (Figure S4B) and Observed Features (Figure S4D), which estimate species richness, displayed narrower distributions and reduced medians in the ASD model group, indicative of decreased microbial richness. Similarly, the Faith PD index (Figure S4C), reflecting phylogenetic diversity, showed a decline in evolutionary breadth among microbial taxa. The Shannon entropy (Figure S4E) and Simpson index (Figure S4F), both integrating richness and evenness, further highlighted reduced community evenness and dominance of fewer taxa in the ASD model group. Collectively, these results suggest a significant contraction in microbial diversity and ecological complexity following VPA induction.

### 2.13. Beta Diversity Analysis of Gut Microbiota

Bray–Curtis Dissimilarity (Figure S5A,B): The Bray–Curtis dissimilarity index, which quantifies compositional differences based on species abundance, exhibited distinct patterns between groups (Figure S5A,B). The control group displayed a narrower distribution and lower median dissimilarity values (Figure S5A), whereas the VPA-induced ASD model group showed a broader distribution and elevated median dissimilarity (Figure S5B). This suggests heightened variability in microbial community structure among ASD model individuals compared to controls.

Unweighted UniFrac Distance (Figure S5C,D): Unweighted UniFrac analysis, which accounts for phylogenetic presence/absence differences, further corroborated these trends. The control group exhibited lower median distances and tighter clustering (Figure S5C), while the ASD model group displayed significantly higher median distances and greater dispersion (Figure S5D). This indicates that microbial lineages in the ASD model group diverged phylogenetically from the control group, independent of abundance variations.

Weighted UniFrac Distance (Figure S5E,F): Weighted UniFrac analysis, incorporating both phylogenetic relationships and species abundance, revealed similar patterns. The control group maintained lower median distances (Figure S5E), whereas the ASD model group showed elevated distances with increased variability (Figure S5F). These results highlight that ASD-associated microbial shifts encompass both phylogenetic and abundance-level alterations, underscoring a multifaceted dysbiosis.

### 2.14. Network Correlation Analysis

The network correlation analysis (Figure S6A–D) revealed a complex interaction pattern among various bacterial families within the microbial community (Figure S6A). Notably, Actinomycetota and Bacillota exhibited numerous interactions, suggesting a high level of co-occurrence and potential synergistic relationships (Figure S6B). Additionally, Bacteroidota and Pseudomonadota also showed significant interactions, indicating their possible functional interdependencies (Figure S6C,D).

The observed interaction patterns provide valuable insights into the structure and function of the microbial community. The frequent interactions between Actinomycetota and Bacillota may reflect their complementary roles in nutrient cycling and environmental adaptation. Similarly, the interactions between Bacteroidota and Pseudomonadota could be indicative of their collaborative efforts in degrading complex organic matter. Further research is warranted to elucidate the specific mechanisms underlying these interactions and their implications for ecosystem health and stability.

## 3. Discussion

This study demonstrates that prenatal valproic acid (VPA) exposure induces autism spectrum disorder (ASD)-like behavioral phenotypes in rats, characterized by social deficits, repetitive grooming, and impaired spatial memory, which co-occurred with gut microbiota dysbiosis and systemic neuroinflammation. By systematically characterizing gut–brain axis (GBA) dysfunction in this model, we establish that disrupted microbial diversity and depletion of short-chain fatty acid (SCFA)-producing taxa (e.g., Clostridia, Lachnospiraceae) constitute central drivers of ASD-like pathophysiology. Specifically, 16S rRNA sequencing revealed significant reductions in α-diversity indices and decreased Firmicutes/Bacteroidetes ratios, while taxonomic profiling identified distinct microbial shifts: depletion of SCFA-producing Clostridia and Lachnospiraceae alongside enrichment of pro-inflammatory Bacteroidia and Enterobacteriaceae.

Building upon these taxonomic alterations observed in Figure 6F—particularly the unique Enterobacteriaceae ASVs and depleted Clostridiaceae ASVs—we propose mechanistic links to ASD pathogenesis. Although strain-specific validation remains ongoing, the identified Enterobacteriaceae ASVs may exacerbate intestinal inflammation through LPS-TLR4/NF-κB signaling [13], whereas depleted Clostridiaceae ASVs could impair epithelial barrier function via reduced butyrate synthesis [14]. These testable hypotheses form the basis of our active metagenomic investigation.

The levels of pro-inflammatory cytokines, including IL-1β and TNF-α, were significantly elevated in the prefrontal cortex, accompanied by microglial activation (increased number of Iba1+ cells). The elevation of these neuroinflammatory markers exhibited a significant negative correlation with the reduction in gut microbiota responsible for producing short-chain fatty acids. These findings align with the hypothesis that gut dysbiosis disrupts SCFA-mediated anti-inflammatory signaling, exacerbating neuroinflammation and oxidative stress (MDA: ↑, GSH: ↓; *p* < 0.0001), ultimately driving ASD-like behavioral impairments. Notably, multivariate analyses revealed strong correlations between Lachnospiraceae abundance and social interaction time, supporting a gut–brain axis mechanism [9]. Behaviorally, VPA-exposed rats exhibited a reduction in social interaction time, an increase in marble-burying frequency, and a prolongation of Morris water maze escape latency, all of which correlated with SCFA-producing taxa (e.g., Clostridia, Lachnospiraceae) [15,16].

These findings suggest a mechanistic cascade where prenatal VPA exposure disrupts the maternal–fetal gut microbiota seeding, leading to postnatal depletion of SCFA-producing taxa [3,10]. The resulting SCFA-producing taxa (e.g., Clostridia, Lachnospiraceae) deficiency likely impairs histone deacetylase (HDAC) regulation, as evidenced by reduced H3K9 acetylation in the IL-1β promoter region, thereby exacerbating neuroinflammation [10]. Concurrently, SCFA-producing taxa (e.g., Clostridia, Lachnospiraceae) depletion compromises the blood–brain barrier integrity, allowing pro-inflammatory cytokines to infiltrate the CNS and trigger microglial activation, a key driver of synaptic pruning deficits in ASD [17,18].

Our results corroborate previous reports linking VPA exposure to ASD-like behaviors and gut dysbiosis in rodents [19,20]. However, unlike studies employing single-dose VPA protocols [10,21], our multi-day dosing regimen (GD11.5–13.5) induced more pronounced microbial shifts, particularly the expansion of Bacteroidia (↑) and depletion of Firmicutes (↓), which may reflect cumulative epigenetic modifications during critical neurodevelopmental windows [22]. Intriguingly, while some studies reported inconsistent behavioral outcomes in VPA models due to variable habituation protocols [23], our standardized behavioral assays (e.g., three-chamber social interaction with 10 min phases) minimized confounding factors, yielding robust deficits in social novelty preference (Stranger 2 interaction: ↓, *p* < 0.0001). Furthermore, the observed elevation of Enterobacteriaceae (↑) aligns with clinical ASD cohorts [24,25,26], yet contrasts with murine VPA models showing dominant Clostridiales enrichment [27], possibly due to species-specific microbial resilience.

Our results align with prior studies highlighting gut dysbiosis in ASD models [8,19,28]. De Theije et al. [29] reported reduced Firmicutes and increased Bacteroidetes in VPA-exposed mice, consistent with our taxonomic shifts. Notably, the timing of microbiota disruption (post-weaning onset) in our longitudinal analysis mirrors the “window of vulnerability” observed in human infants, where gut microbiota maturation delays correlate with ASD risk [30,31]. The SCFA-producing taxa (e.g., Clostridia, Lachnospiraceae) depletion phenotype also echoes findings in ASD patients, who exhibit lower fecal butyrate levels and reduced Lachnospiraceae abundance [32,33,34].

Contrasts with some studies may arise from model parameters. For example, our failure to detect Proteobacteria enrichment differs from the C57BL/6 mouse model [35], possibly due to strain-specific microbiota resilience or VPA dosing schedule (gestational days 11.5–13.5 vs. single-dose exposure). Additionally, the strong correlation between SCFA-producing taxa (e.g., Clostridia, Lachnospiraceae) depletion and microglial activation underscores direct metabolic–neuroimmune interactions over intestinal barrier dysfunction, supporting the “SCFA insufficiency hypothesis” proposed by Wang et al. [36].

This study advances the GBA theory by establishing a temporal and causal link between microbial dysbiosis, SCFA-producing taxa (e.g., Clostridia, Lachnospiraceae), and neurobehavioral abnormalities. The discovery that SCFA-producing taxa (e.g., Clostridia, Lachnospiraceae) precede behavioral onset suggests a critical need for early intervention. Mechanistically, we demonstrate that SCFA-mediated HDAC inhibition regulates neuroinflammatory pathways, providing a molecular explanation for the therapeutic effects of HDAC inhibitors in preclinical ASD models [37,38].

Our study advances the “gut–brain axis” hypothesis in ASD by mechanistically linking prenatal environmental insults to neurodevelopmental outcomes via microbial-metabolite pathways [39]. The depletion of SCFA-producing taxa (e.g., Clostridia, Lachnospiraceae) and subsequent reduction in butyrate levels (inferred from PICRUSt2 predictions) may impair blood–brain barrier integrity and exacerbate neuroinflammation through NF-κB activation [40,41]. Practically, these findings highlight microbiota modulation (e.g., probiotics targeting Lachnospiraceae) and SCFA supplementation as viable therapeutic strategies. For instance, butyrate administration has been shown to ameliorate social deficits in ASD models [42], suggesting translational potential for clinical trials.

## 4. Materials and Methods

### 4.1. Experimental Design and Animal Procedures

All procedures were conducted in accordance with the Institutional Animal Care Committee guidelines of Guangzhou Institute of Biomedicine and Health (Approval No. IACUC-2022-0063) under AAALAC-accredited SPF conditions.

#### 4.1.1. Animal Housing and Breeding

Adult Sprague–Dawley rats (18 females, 9 males; initial body weight 200–250 g) were housed in a controlled environment maintained at 22 ± 1 °C with 55 ± 5% relative humidity and a 12 h light/dark cycle (lights on at 07:00). Animals received autoclaved water and standard rodent chow ad libitum. For mating, male and female rats were co-housed at 17:00, with vaginal plug verification performed at 09:00 the following morning. Plug-positive females were designated as gestational day (GD) 0.5.

#### 4.1.2. Prenatal VPA Exposure

Twenty-four confirmed pregnant dams were randomly assigned to two groups via stratified randomization. From GD11.5 to GD13.5 (corresponding to peak neocortical neurogenesis in rats), the VPA-exposed group (*n* = 12 dams) received daily intraperitoneal injections of sodium valproate (400, 450, and 400 mg/kg on GD11.5, 12.5, and 13.5, respectively) dissolved in endotoxin-free saline. Control dams (*n* = 6) received equivalent volumes of 0.9% NaCl. Male offspring born to VPA-exposed dams are hereafter designated as “prenatal VPA-exposed offspring”.

#### 4.1.3. Postnatal Group Allocation

Male offspring were cross-fostered to eliminate litter effects and randomly assigned at postnatal day 21 (PND21) to two experimental groups: Control group (*n* = 12, saline-treated offspring from control dams) and VPA group (*n* = 12, male offspring from VPA-exposed dams). Experimenters were blinded to group assignments throughout behavioral testing and biochemical analyses. Behavioral tests were conducted, then histological evaluations were performed.

This study is reported in accordance with the ARRIVE guidelines [43]. All experimental procedures were approved by the Institutional Animal Care and Use Committee (IACUC) of the Guangzhou Institute of Biomedicine and Health, Chinese Academy of Sciences (Ethics Approval No. CAS (IACUC:2023081)), and strictly adhered to the Guide for the Care and Use of Laboratory Animals [44]. Euthanasia and anesthesia protocols followed the 2020 American Veterinary Medical Association (AVMA) Guidelines for the Euthanasia of Animals, and no prohibited methods (e.g., chloral hydrate, ether, or chloroform) were employed. Every effort was made to minimize animal suffering, including the use of appropriate analgesic regimens and humane endpoints during the study.

### 4.2. Behavioral Tests

SD rats were placed in the experimental room one hour prior to the procedures to acclimate to the laboratory conditions.

(1) Three-chamber social interaction test

We assessed sociability and social novelty preference using a rectangular polycarbonate arena (120 × 40 × 40 cm) divided into three equal compartments by retractable doors. Prior to formal testing, rats underwent a 10-day habituation protocol. From days 1 to 7, animals were acclimated daily for 2 h to testing conditions (23 °C, 50 lux illumination). During days 8–10, graduated exposure was implemented, beginning with 3 min of central compartment exploration followed by 3 min of full arena access each day.

Experimental Sequence: All behavioral assessments were conducted between 09:00 and 12:00 during the animals’ active phase. The test comprised two sequential phases:

Sociability Test (0–10 min): Following a 5 min baseline period with empty wire cages in both lateral chambers, an age-/sex-matched Sprague–Dawley “Stranger 1” rat was introduced into one lateral cage while the opposite chamber retained an empty control cage. Social exploration behavior (sniffing, following, physical contact) directed toward Stranger 1 vs. the empty cage was video-recorded.

Social Novelty Preference (10–20 min): A novel “Stranger 2” rat replaced the empty cage, while Stranger 1 remained in its original location. The time spent interacting with the familiar Stranger 1 versus the novel Stranger 2 was quantified to assess social novelty discrimination.

(2) Self-grooming test

Rats were individually placed in clean polycarbonate cages under 50 lux illumination. After 10 min habituation, spontaneous grooming behaviors (facial cleaning, body licking, genital/tail grooming) were video-recorded for 10 min using a Logitech C920 HD Pro webcam (manufactured by Logitech International S.A., headquartered in Lausanne, Switzerland).

(3) Marble-Burying Test

SD rats were individually placed in polycarbonate test chambers containing 5 cm of fresh corncob bedding. After a 3 min acclimatization period, animals were temporarily transferred to holding cages. Sixteen black glass marbles (1.6 mm diameter) were arranged in a standardized 4 × 4 grid pattern spaced 3 cm apart. Subjects were returned to the chamber for a 10 min testing session under 50 lux illumination. Sessions were video-recorded from orthogonal angles.

(4) Open field test

Behavioral assessments were conducted in a square open-field arena measuring 100 × 100 × 40 cm (L × W × H), which featured a clearly demarcated central zone (50 × 50 cm). Animal movement throughout the arena was continuously recorded using an overhead camera system for subsequent analysis. Following a 10 min habituation period during which animals were allowed to freely explore the novel environment, formal behavioral testing commenced for a duration of 10 min. Throughout this testing phase, we quantified specific exploratory and anxiety-related behaviors using the Noldus EthoVision XT software.

(5) Morris Water Maze Behavioral Assessment

Spatial learning and memory were assessed in a Morris water maze consisting of a black circular pool (150 cm diameter) filled with temperature-controlled water (23.0 ± 0.5 °C), opacified using nontoxic white tempera paint. A hidden escape platform (15 cm diameter) was submerged 1.5 cm below the surface in the target quadrant. Four high-contrast geometric patterns provided consistent distal spatial cues under uniform illumination (50 ± 5 lux). Animal movements were tracked using an overhead camera system (EthoVision XT v15.0, Noldus Information Technology, Wageningen, The Netherlands) with positional accuracy validated at <2% error via chessboard calibration prior to testing.

During the 5-day acquisition phase, animals completed four daily trials (90 s maximum/trial) with 25 min inter-trial intervals. Starting points (north, south, east, west) were randomized across trials. Animals failing to locate the platform within 90 s were manually guided to it and remained there for 10 s for spatial orientation. Twenty-four hours after the final acquisition session, spatial memory was evaluated in a 60 s probe trial where the platform was removed. All behavioral parameters were quantified from video recordings using EthoVision XT software.

### 4.3. Sacrificing Animals and Preparing Tissues

Animals were sacrificed after completion of behavioral tests. Anesthesia was induced by intraperitoneal administration of ketamine (100 mg/kg) and xylazine (10 mg/kg). For biochemical analyses, rats (*n* = 12 per group) were decapitated without perfusion. The brains were rapidly removed, and the prefrontal cortex and hippocampus were dissected on ice, snap-frozen in liquid nitrogen, and stored at −80 °C until assayed. For histological evaluations, rats (*n* = 6 per group) were transcardially perfused with 0.9% NaCl followed by fixation with 4% paraformaldehyde (PFA, Merck, Germany). After careful removal of the brain, it was post-fixed in 4% PFA for 24 h. The hippocampus and prefrontal cortex were subsequently isolated, processed, and paraffin-embedded.

### 4.4. Biochemical Analysis

The levels of oxidative stress markers (GSH, T-SOD, GSH-PX, MDA, CAT, T-NOS, NO) and pro-/anti-inflammatory cytokines (IL-6, IL-10, IL-1β, TNF-α) were quantified using commercial kits (Nanjing Jiancheng Institute of Biotechnology, Nanjing, China) following the manufacturers’ guidelines.

#### 4.4.1. Sample Preparation

Prefrontal cortex tissues were homogenized in ice-cold physiological saline (1:9 *w*/*v*) and centrifuged at 3000× *g* for 10 min at 4 °C. Supernatants were aliquoted and stored at −80 °C until analysis.

#### 4.4.2. Cytokine Quantification (ELISA)

IL-1β, IL-6, TNF-α, and IL-10 levels were measured via sandwich ELISA. Briefly, 10 µL of supernatant was diluted 1:5 with sample diluent (40 µL), added to antibody-coated wells with HRP-conjugated detection antibody (100 µL), and incubated at 37 °C for 60 min. After 5 washes, TMB substrate (50 µL each of Solutions A/B) was added. The reaction was terminated with 50 µL of stop solution (H_2_SO_4_), and absorbance was read at 450 nm within 15 min. Concentrations were interpolated from standard curves (0–120 pg/mL for IL-1β; R^2^ > 0.99).

#### 4.4.3. Oxidative Stress Marker Analysis

T-NOS: Double-antibody ELISA (detection range: 25–400 ng/mL).

NO: Quantified indirectly as nitrite via Griess reaction at 540 nm.

GSH: DTNB reduction assay at 412 nm.

SOD: Inhibition of nitroblue tetrazolium reduction at 550 nm.

MDA: Thiobarbituric acid reactive substances (TBARS) method at 532 nm.

CAT: H_2_O_2_ decomposition kinetics at 240 nm.

All measurements were performed in duplicate and normalized to total protein concentration (BCA assay, Beyotime, Shanghai, China).

### 4.5. 16S rRNA Sequencing and Bioinformatics Analysis

- Sample Collection and Preservation

Fresh fecal samples were collected from experimental rats using aseptic procedures after immobilization:

(a) The rat tail was gently lifted, and sterile finger cot pressure was applied to the lower abdomen to promote defecation.
(b) Fecal pellets were transferred into pre-labeled 2 mL sterile centrifuge tubes (Axygen, PCR-02-C, Corning Incorporated, Union City, CA, USA) using disposable sterile forceps.
(c) Each tube contained 3–5 intact fecal pellets (approximately 100 mg).
(d) Sampling was consistently performed between 09:00 and 11:00 daily to eliminate circadian rhythm effects on gut microbiota.
(e) Samples were immediately snap-frozen in liquid nitrogen (<5 min), stored at −80 °C, and transported to the testing facility on dry ice.

B. DNA Extraction and 16S rRNA Gene Amplification

(1) Genomic DNA Extraction Using Modified CTAB Method

Tissue homogenization was performed with 0.1 mm zirconium beads (BioSpec Products, 11079101z, Bartlesville, OK, USA) under liquid nitrogen pre-cooling using a FastPrep-24 homogenizer (MP Biomedicals, Solon, OH, USA).

DNA purity criteria: NanoDrop 2000 (Thermo Fisher Scientific, Waltham, MA, USA) absorbance ratios A260/A280 = 1.8–1.9, A260/A230 > 2.0. Qualified DNA was diluted to 1 ng/μL using sterile ultrapure water (Millipore Milli-Q, Merck KGaA, Darmstadt, Germany).

(2) PCR Amplification of V3-V4 Regions

The V3-V4 hypervariable regions were amplified using primers 341F (5′-CCTAYGGGRBGCASCAG-3′) and 806R (5′-GGACTACNNGGGTATCTAAT-3′).

PCR conditions:

Template: Diluted genomic DNA

Primers: Barcode-labeled specific primers

Enzyme: Phusion^®^ High-Fidelity PCR Master Mix with GC Buffer (New England Biolabs, Ipswich, MA, USA)

Thermal cycling: 98 °C for 1 min; 30 cycles (98 °C for 10 s, 50 °C for 30 s, 72 °C for 30 s); 72 °C for 5 min.

C. Library Preparation and High-Throughput Sequencing

(1) Product Purification

Amplified products (~550 bp) were verified by 2% agarose gel electrophoresis (1× TAE buffer) and purified using the TIANgel Midi Purification Kit (Tiangen, Beijing, China, DP209).

(2) Library Construction

Libraries were prepared with the NEBNext Ultra II DNA Library Prep Kit (Illumina, San Diego, CA, USA, E7645S). Quality control included fragment distribution analysis (Agilent 5400 Bioanalyzer, Agilent Technologies, Santa Clara, CA, USA; target peak: 450–650 bp) and quantification (Qubit 4.0, Thermo Fisher; concentration >20 nM).

(3) Illumina Sequencing

Sequencing was performed on the NovaSeq 6000 platform (Illumina, San Diego, CA, USA) using PE250 mode, with a depth of 50,000 reads/sample.

D. Bioinformatics Analysis

(1) Raw Data Processing

QIIME2 (v2023.2) pipeline: DADA2 denoising, chimera removal, and ASV table generation. Taxonomic annotation utilized the SILVA 138.1 database (V3-V4 region extraction).

(2) Diversity Analysis

α-diversity: Chao1, Shannon, and Faith’s PD indices (QIIME2 core-metrics).

β-diversity: Bray–Curtis and UniFrac distances visualized via PCoA/NMDS.

(3) Differential Species Identification

Multi-method validation: LEfSe (LDA score > 2.0), ANCOM (W-statistic > 0.7), DESeq2 (FDR < 0.05).

(4) Statistical Analysis

Co-occurrence networks: Constructed in Gephi (v0.10.1; Spearman |ρ| > 0.6, *p* < 0.01).

Multivariate analysis: RDA (vegan package) and PLS-DA (mixOmics package) in R (v4.3.1) using the “vegan” (v2.6-4) and “mixOmics” (v6.24.0) packages, respectively.

### 4.6. Immunofluorescence Staining

Brain sections were deparaffinized and subjected to antigen retrieval. After three 5 min washes with phosphate-buffered saline (PBS, pH 7.4), non-specific binding sites were blocked with 4% (*w*/*v*) bovine serum albumin (BSA) in PBS containing 0.3% Triton X-100 (PBST) for 1 h at room temperature (RT). Sections were incubated with rabbit primary antibodies (1:200 dilution in blocking solution) targeting specific antigens at 4 °C for 16 h in a humidified chamber. Following PBST washes (3 × 10 min), samples were treated with Alexa Fluor 488-conjugated goat anti-rabbit IgG secondary antibodies in the dark at RT for 1 h. Nuclei were counterstained with 4′, 6-diamidino-2-phenylindole (DAPI; 1 μg/mL) for 10 min in darkness.

Confocal imaging was performed using a laser scanning microscope (Leica TCS SP8 STED 3X, Leica Microsystems, Wetzlar, Germany) with the following standardized parameters: Objective: 63× oil immersion (HC PL APO CS2; NA = 1.40); laser lines: 488 nm (Alexa Fluor 488, Thermo Fisher Scientific, Waltham, MA, USA; 15% power), 405 nm (DAPI; 8% power); detection windows: 500–550 nm (green channel), 410–480 nm (blue channel); pinhole: 1.0 Airy unit (optical section thickness: 0.8 μm); scan mode: Sequential line scanning (frame averaging: 3×; scan speed: 400 Hz); Z-stack: Step size 0.5 μm (total depth: 8–10 μm per stack); and image resolution: 1024 × 1024 pixels (pixel size: 120 nm). Detector gains were calibrated daily using control sections to avoid saturation (max pixel intensity <4095 AU). Z-stack images were processed for maximum intensity projection and analyzed for integrated density (IntDen) using ImageJ (Fiji v2.3.0) with background subtraction (rolling ball radius: 50 pixels).

### 4.7. Statistical Analysis

Statistical analyses were performed using GraphPad Prism software (version 9.0; GraphPad Software, San Diego, CA, USA). Continuous data are presented throughout the figures and text as mean ± standard deviation (SD). Normality of data distribution was assessed using the Shapiro–Wilk test. For comparisons between the two experimental groups, an unpaired two-tailed Student’s *t*-test was employed when data met assumptions of normality and homogeneity of variance. Where data violated normality assumptions, the non-parametric Mann–Whitney U test was used instead. Statistical significance was defined as a *p*-value less than 0.05 for all analyses.

## 5. Conclusions

This study establishes that prenatal valproic acid (VPA) exposure induces gut microbiota dysbiosis characterized by reduced microbial diversity and depletion of SCFA-producing taxa (e.g., Clostridia, Lachnospiraceae), concomitant with neuroinflammation and oxidative stress that collectively drive ASD-like phenotypes in rodents. Through integrated behavioral, microbial, and molecular analyses, we provide mechanistic evidence linking gut dysbiosis to neurodevelopmental deficits via impaired SCFA-mediated pathways, highlighting microbiota modulation and SCFA supplementation as promising therapeutic strategies for ASD.

While these correlative relationships are robustly demonstrated, causal validation through fecal microbiota transplantation or SCFA restitution experiments remains essential. Notably, existing intervention studies demonstrate that rectifying similar dysbiotic signatures via probiotics [45,46] or FMT [47,48] ameliorates behavioral deficits in ASD models, supporting the therapeutic potential of our findings. Future work should prioritize: (i) causal validation of identified dysbiotic signatures; (ii) clinical translation of microbial and metabolic biomarkers; and (iii) multi-omics dissection of host–microbe crosstalk to evaluate targeted interventions across preclinical and human cohorts. The robust phenotypic-microbial correlations established here provide both actionable biomarkers for ASD risk stratification and a mechanistic foundation for microbiota-directed therapeutics.

## Data Availability

All datasets generated and analyzed during this study are presented in this published article.

## Supplementary Materials

The following supporting information can be downloaded at: https://www.mdpi.com/article/10.3390/ijms26188931/s1.
