# Supplementary material for: Altered Gut Microbial Diversity and Depletion of SCFA-Producing Taxa Associated with ASD-like Phenotypes in a Prenatal VPA Rat Model
Source: Int J Mol Sci. 2025 Sep 13;26(18):8931. doi: 10.3390/ijms26188931 (PMC12469871; doi:10.3390/ijms26188931)
Supplement: Supplementary file 1 [file ijms-26-08931-s001.zip › ijms-3826942-supplementary.pdf]

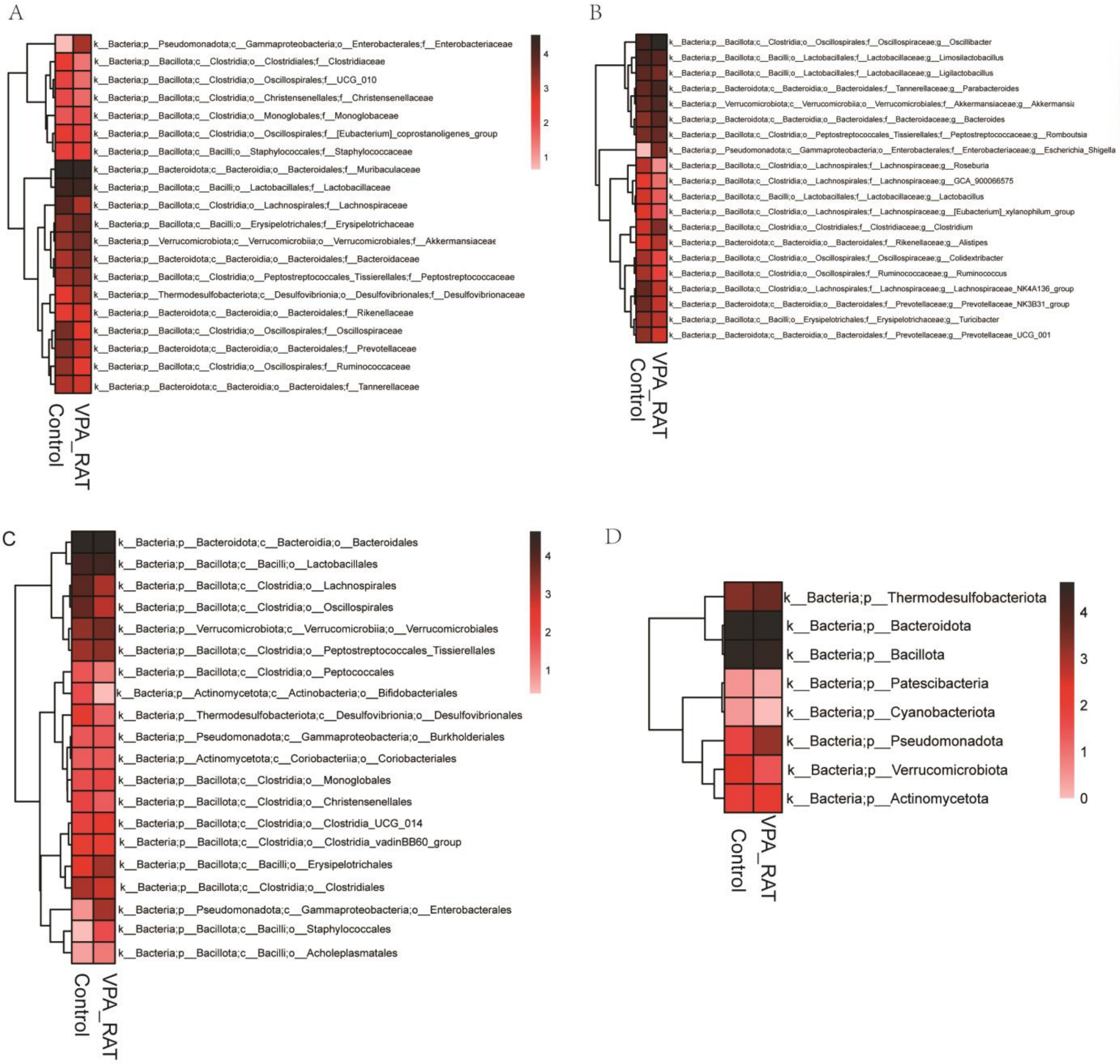

Figure S1. Taxonomic profiling of gut microbiota in control and VPA-induced ASD model rats. (A) Family-level analysis. (B) Genus-level alterations. (C) Order-level differences. (D) Phylum-level composition.

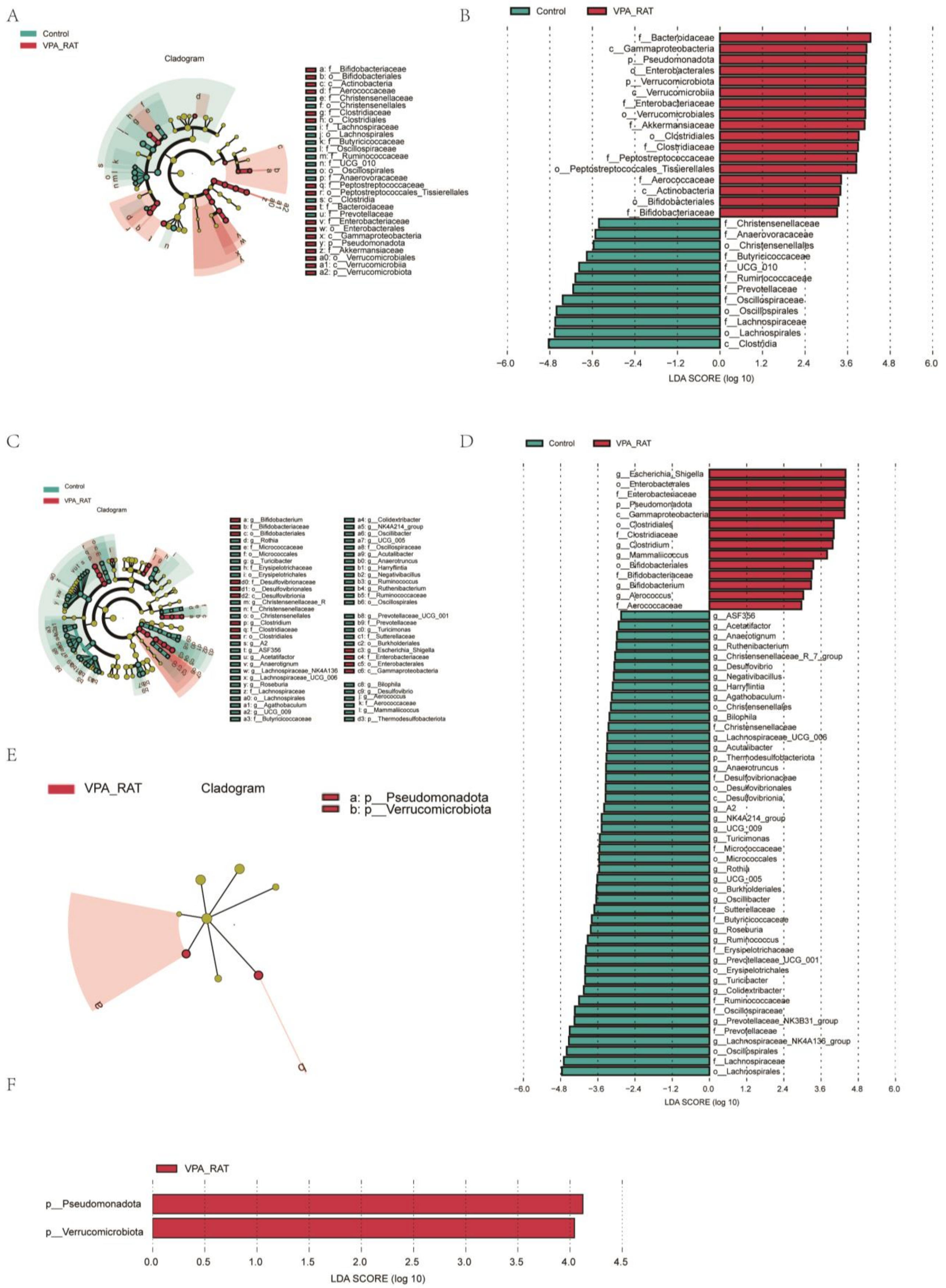

Figure S2. Comparative gut microbiota analysis between the control group and VPA-induced ASD rat models. (A) Family-level cladogram. (B) Family-level LDA Score. (C) Genus-level cladogram. (D) Genus-level LDA Score. (E) Phylum-level cladogram. (F) Phylum-level LDA Score.

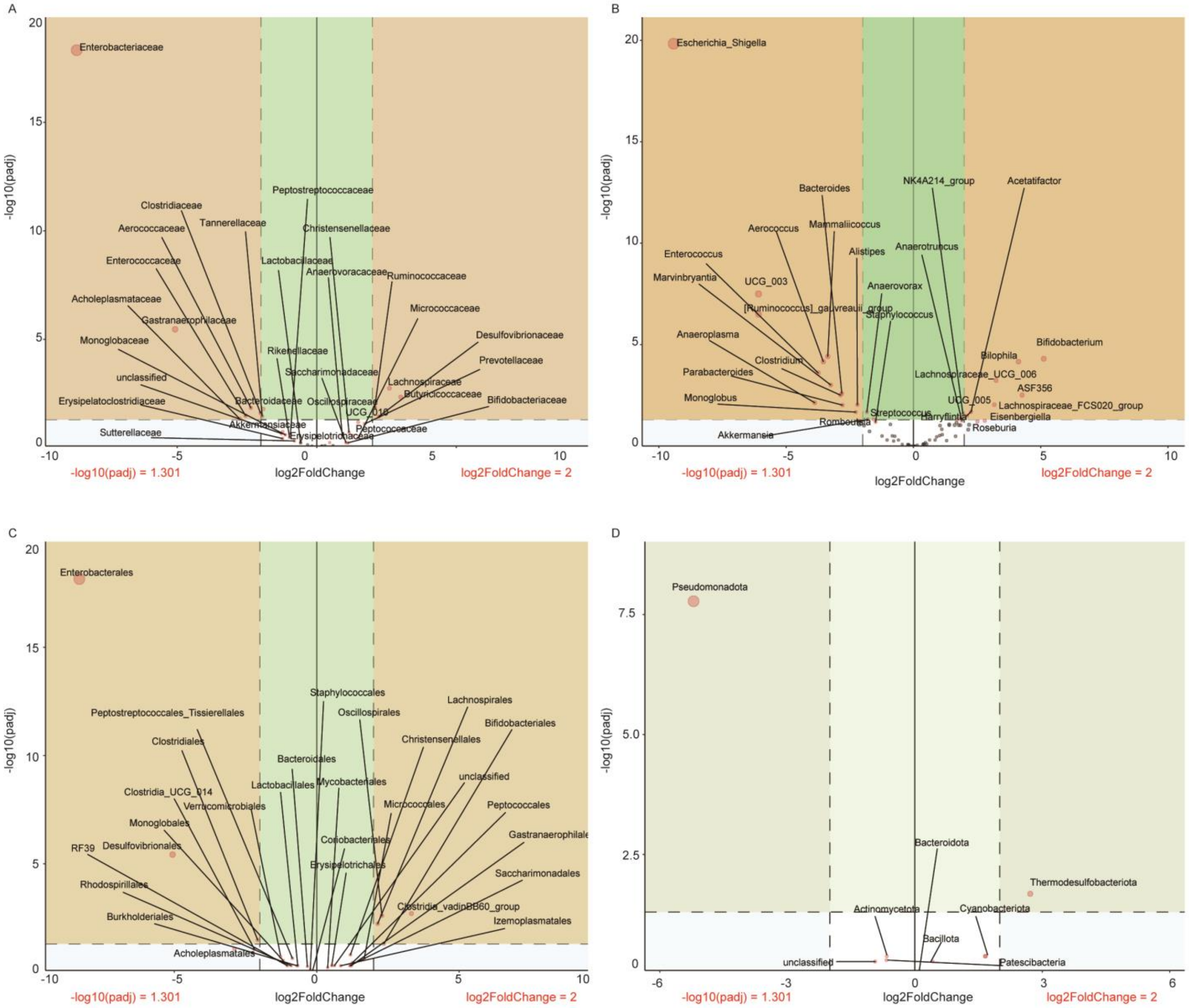

Figure S3. Differential gut microbiota composition between Control and VPA-induced ASD model rats. (A) Family-level analysis. (B) Genus-level analysis. (C) Order-level analysis. (D) Phylum-level analysis.

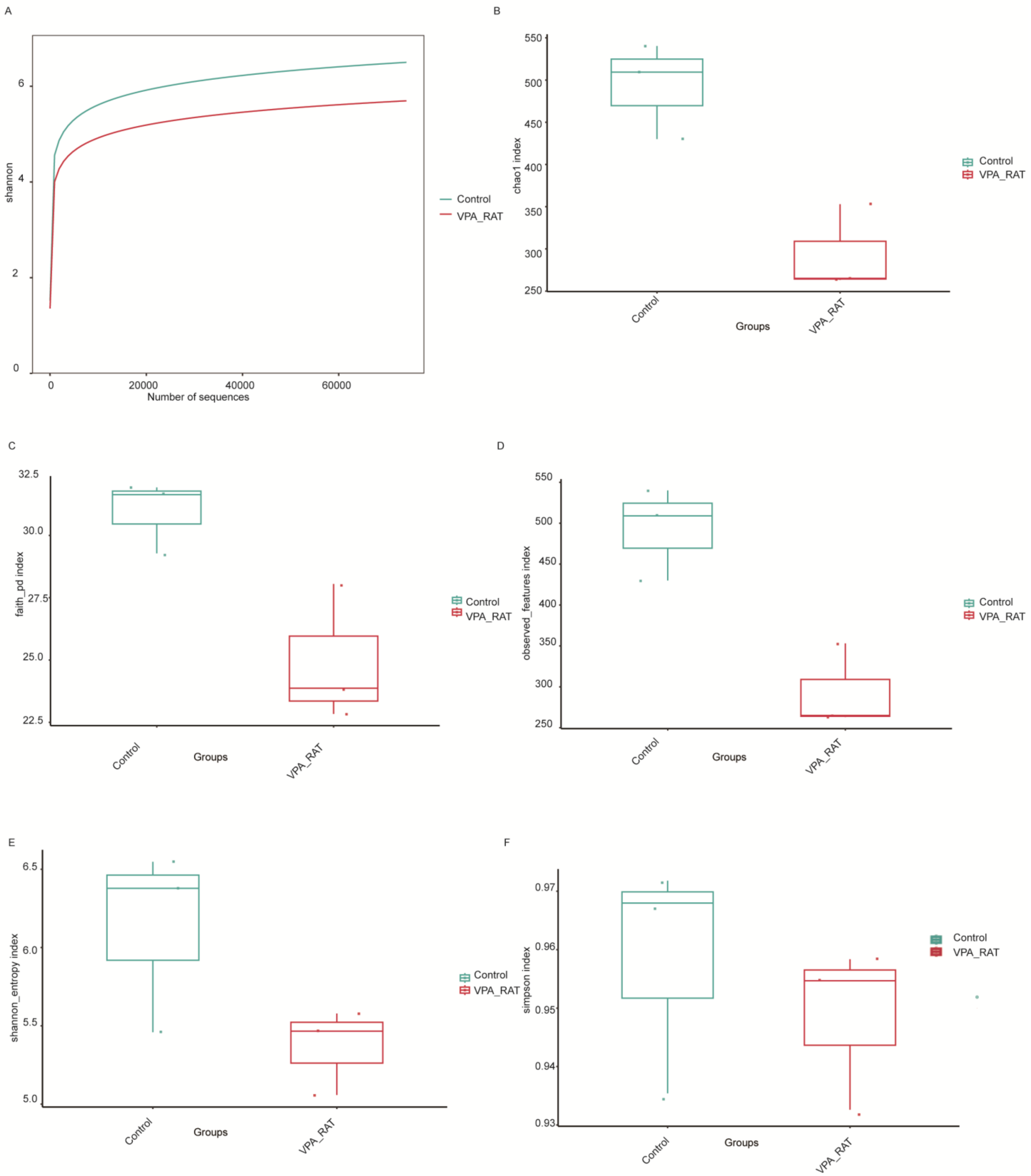

Figure S4. Gut microbiota alpha diversity in control and VPA-exposed offspring. (A) Shannon rarefaction curves. (B–F) Boxplots of alpha diversity indices: (B) Chao1, (C) Faith PD, (D) Observed Features, (E) Shannon, (F) Simpson. Blue: control group; red: VPA group. All boxplots show median ± IQR.

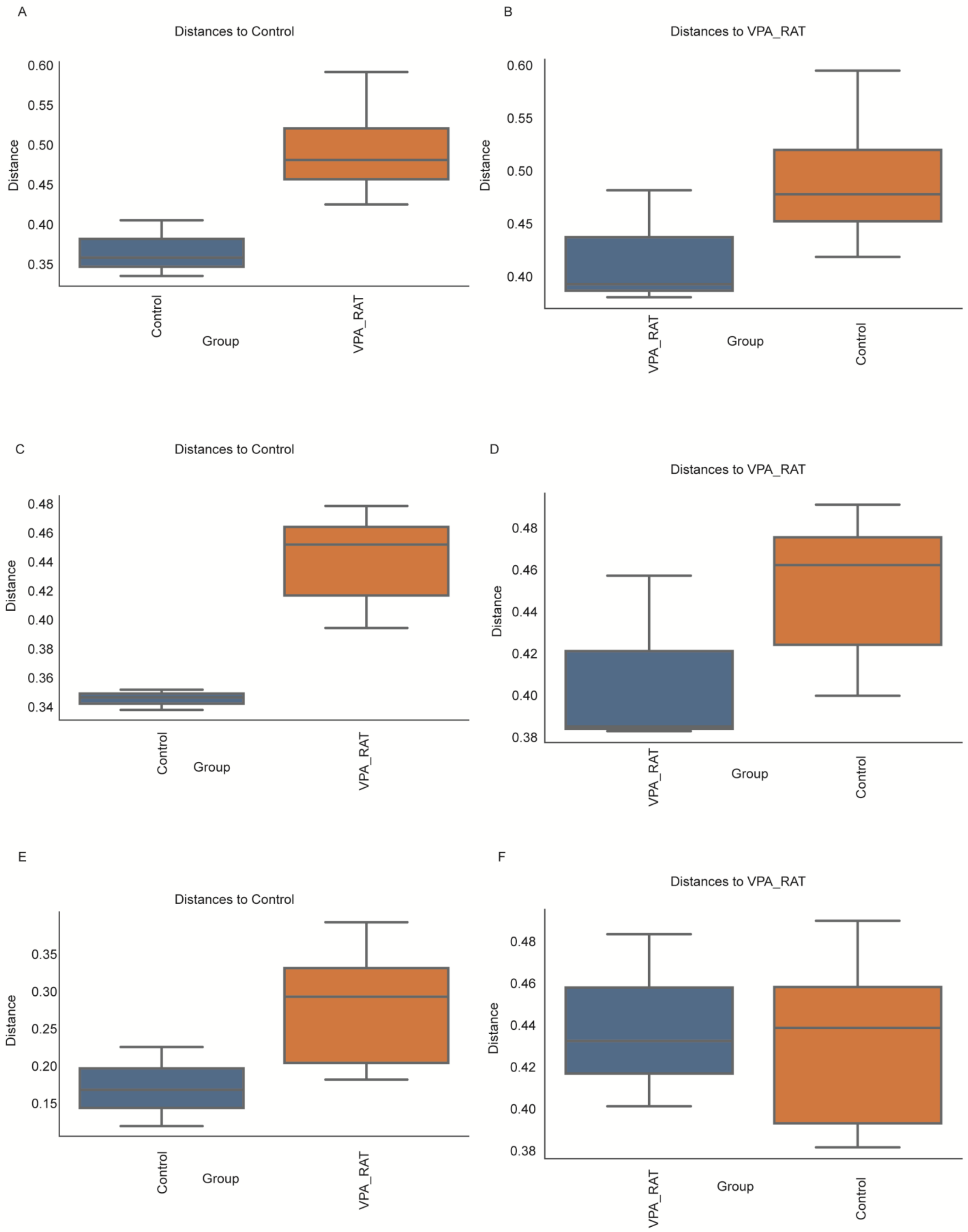

Figure S5. Beta diversity analysis of gut microbiota. (A) Bray–Curtis dissimilarity (Distance to control group). (B) Bray–Curtis (Distance to VPA group). (C) Unweighted UniFrac (Distance to control group). (D) Unweighted UniFrac (Distance to VPA group). (E) Weighted UniFrac (Distance to control group). (F) Weighted UniFrac (Distance to VPA group).

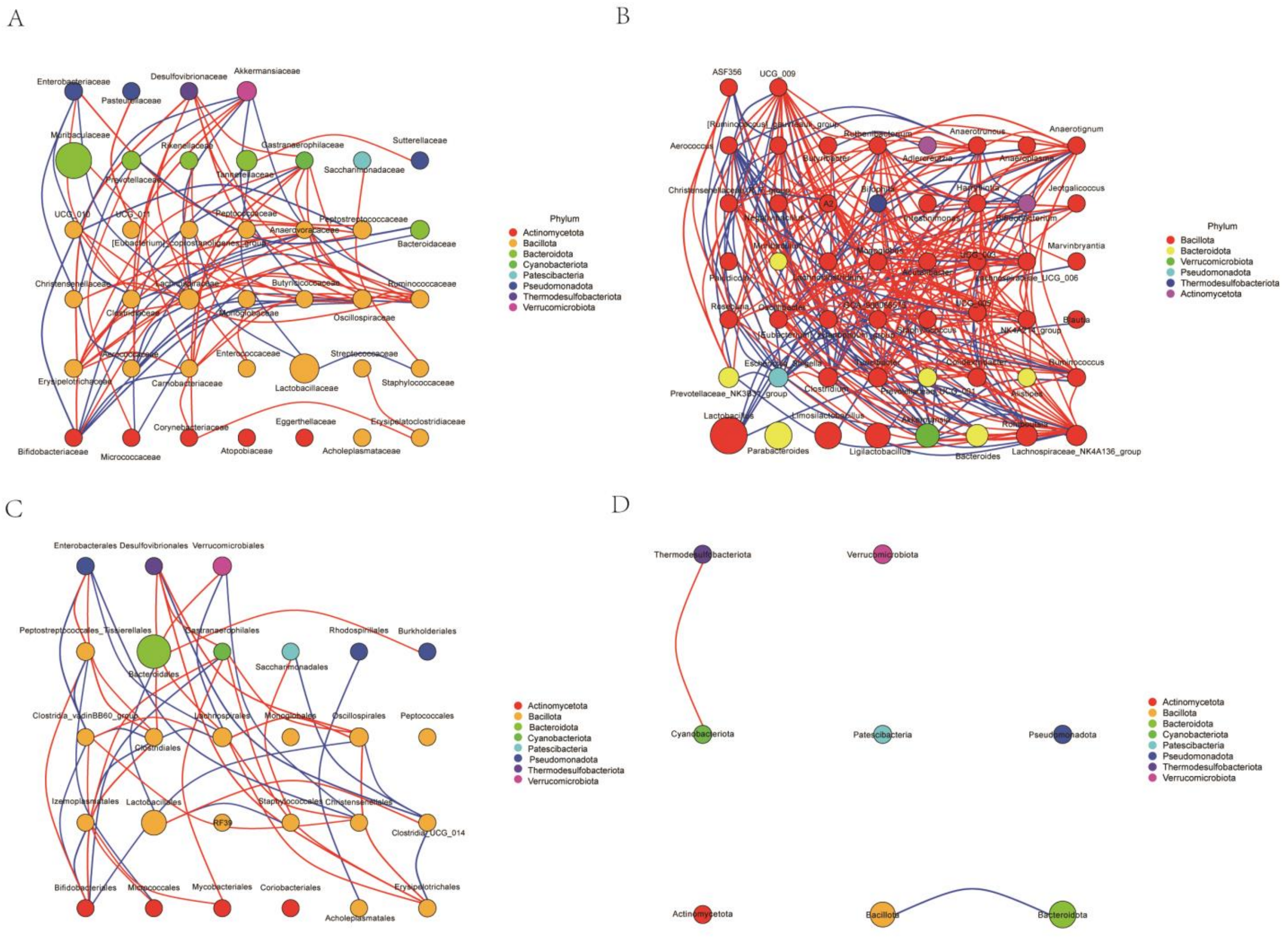

Figure S6. Network correlation analysis. (A) Family-level network correlation analysis. (B) Genus-level network correlation analysis. (C) Order-level network correlation analysis. (D) Phylum-level network correlation analysis.
